# Theory of mind in women with borderline personality disorder or schizophrenia: differences in overall ability and error patterns

**DOI:** 10.3389/fpsyg.2015.01239

**Published:** 2015-08-24

**Authors:** Anja Vaskinn, Bjørnar T. Antonsen, Ragnhild A. Fretland, Isabel Dziobek, Kjetil Sundet, Theresa Wilberg

**Affiliations:** ^1^Department of Psychology, University of Oslo, Oslo, Norway; ^2^NORMENT KG Jebsen Centre for Psychosis Research, Oslo University Hospital, Oslo, Norway; ^3^Institute for Clinical Medicine, University of Oslo, Oslo, Norway; ^4^Department of Personality Psychiatry, Clinic of Mental Health and Addiction, Oslo University Hospital, Oslo, Norway; ^5^Berlin School of Mind and Brain, Humboldt University Berlin, Berlin, Germany; ^6^Department of Research and Development, Clinic of Mental Health and Addiction, Oslo University Hospital, Oslo, Norway

**Keywords:** borderline personality disorder, schizophrenia, social cognition, theory of mind, mentalizing

## Abstract

Although borderline personality disorder (BPD) and schizophrenia (SZ) are notably different mental disorders, they share problems in social cognition—or understanding the feelings, intentions and thoughts of other people. To date no studies have directly compared the social cognitive abilities of individuals with these two disorders. In this study, the social cognitive subdomain theory of mind was investigated in women with BPD (*n* = 25), women with SZ (*n* = 25) and healthy women (*n* = 25). An ecologically valid video-based measure (Movie for the Assessment of Social Cognition) was used. For the overall score, women with SZ performed markedly below both healthy women and women with BPD, whereas women with BPD did not perform significantly different compared to the healthy control group. A statistically significant error type × group interaction effect indicated that the groups differed with respect to kind of errors. Whereas women with BPD made mostly overmentalizing errors, women with SZ in addition committed undermentalizing errors. Our study suggests different magnitude and pattern of social cognitive problems in BPD and SZ.

## Introduction

Impairments in the ability to infer the thoughts, emotions and intentions of others—or social cognition—are present in a number of mental disorders. Difficulties in understanding social information are a diagnostic criterion for autism spectrum disorder (American Psychiatric Association, 2013), and social cognition impairment is central to schizophrenia (SZ; Savla et al., 2013). It is present in affective disorders such as depression (Kohler et al., 2011) and bipolar disorder (Kohler et al., 2011; Samamé et al., 2012) and in personality disorders (Herpertz and Bertsch, 2014) such as borderline personality disorder (BPD; Roepke et al., 2013). Terminology is somewhat different depending on the mental disorder in question. Within the BPD research literature the term mentalizing is often utilized, although the term social cognition is catching on. A distinction has been made between emotional empathy and cognitive empathy with the latter also being known as theory of mind (ToM). Within the SZ research field the preferred term is social cognition, referring to four core domains (Pinkham, 2014), namely emotion processing, social perception, ToM/mental state attribution, and attributional style/bias.

Borderline personality disorde is characterized by disturbed interpersonal relations—together with affective instability, impulsivity, and identity problems (American Psychiatric Association, 2013). Research on disturbed relatedness has been limited compared to other aspects of the disorder. It is possible that interpersonal dysfunction can be an effect of aberrations in social cognition. Results from research on social cognition in BPD have been inconsistent (Roepke et al., 2013). Early research using projective tests provided evidence of an attributional style where individuals with BPD to a larger extent than other people perceive others as malevolent (Westen, 1990). Some studies have found increased performance compared to healthy controls on tasks measuring social perception (Frank and Hoffman, 1986), ToM (Fertuck et al., 2009), and facial emotion perception (Lynch et al., 2006). Such findings led to the claim that individuals with BPD have a “hypervigilant” mind with superior abilities in picking up on social cues. On the other hand, there are also diverging reports where BPD performance is below that of healthy control participants, both for facial emotion perception (Bland et al., 2004) and ToM (Sharp et al., 2011). Roepke et al. (2013) suggest that some of the inconsistencies in results can be attributed to the fact that more complex and ecologically valid tasks are necessary to tease out the social cognition problems of BPD. Individuals with BPD may have particular difficulties with more complex emotional recognition tasks reflecting deficits in higher order integration of social information (Minzenberg et al., 2006; Dyck et al., 2009). One study (Preißler et al., 2010) found individuals with BPD to be impaired compared to healthy controls on a psychometrically sound, complex and ecologically valid test (“Movie for the Assessment for Social Cognition”—MASC; Dziobek et al., 2006) but not on a simpler ToM task (“Reading the mind in the eyes”; Baron-Cohen et al., 2001).

The MASC test is a good candidate for studies that seek to increase the knowledge of social cognition in BPD. In addition to its high ecological validity, it has another advantage by classifying the incorrect responses. The usual right/wrong dichotomy is bypassed through the discrimination between three error types: overmentalizing (over-interpretative mental state reasoning), reduced ToM (insufficient mental state reasoning), and no ToM (lack of mental state concept). This classification enables a more detailed investigation of the nature of mentalizing problems than does the right/wrong ToM dichotomy. In a study of inpatient adolescents that used the MASC test, the association between borderline traits and impaired ToM was driven by a very strong correlation with overmentalizing errors (Sharp et al., 2011). Using more complex and nuanced measures of ToM may increase our understanding of the particular difficulties in social cognition characteristic of BPD. The different error types may also be helpful in distinguishing clinical subgroups from each other in terms of social cognitive impairments.

Indeed, a second recommendation thought to bring the field forward is to conduct comparisons with other clinical populations. SZ is for several reasons a potent comparison group. We are not aware of any comparative ToM studies of SZ and BPD, except for a small study from a forensic setting that found SZ participants to be impaired on simpler ToM tests compared to a combined personality disorder group (Murphy, 2006). Clearly, BPD and SZ are very different disorders. Characteristic features of BPD are instability in self-image, affectivity and interpersonal relations, whereas reality distortion, social withdrawal, neuropsychological and brain abnormalities are important characteristics of SZ. However, the two disorders are not always easy distinguishable in severe states, and they do share problems in relating to other people. Social cognitive impairments are among the central characteristics of SZ (Savla et al., 2013) with large deficits compared to healthy controls for all domains. In SZ, social cognition mediates the association between neurocognition and functional outcome (Schmidt et al., 2011) and is a stronger predictor of functioning than neurocognition—this seems to be especially so for ToM (Fett et al., 2011). Since interpersonal problems are common to the two disorders, and such problems probably, at least to some extent, are associated with problems in perceiving and understanding social communication, i.e., social cognition, it is of interest to investigate this construct in detail across the two disorders. For example, it may provide information on how to better differentiate between the disorders in severe states.

In the current study we explore social cognition, defined as ToM, in women with BPD and in women with SZ. Healthy female control participants (HC) were included as a comparison group. Men and women seem to be differently affected by SZ and BPD, respectively. There are some data to suggest that women with SZ may have a better course of illness than men, with better functioning (Cotton et al., 2009), less pronounced cognitive deficits (Vaskinn et al., 2011) and better social cognition (Scholten et al., 2005; Vaskinn et al., 2007), even though diverging evidence does exist (Ochoa et al., 2012). For BPD, although women experience more symptomatology overall (especially for anxious, depressive, and relational problems), gender differences are small, often less pronounced than in the general population, and the genders are equally disabled by their symptoms (Silberschmidt et al., 2015). Should we find ToM impairments in the current study, they can be expected to be present also in men with these two disorders, possibly to an even larger extent in men with SZ. We have two main aims: First, we are interested to see whether group differences are present for overall ToM abilities across the three groups. Based on the literature we expect participants with BPD to be impaired compared to HC, but not to the same extent, as will be the case for women with SZ. Second, we investigate the distribution of error scores, asking whether there are significant group differences in the types of errors made by participants with BPD or SZ, respectively. We have no specific hypotheses regarding group differences, but expect based on our previous work (Fretland et al., 2015) that women with SZ will commit both under- and overmentalizing errors, and, based on Sharp et al.’s (2011) study reviewed above, that women with BPD will make overmentalizing errors.

## Materials and Methods

### Participants

The study was conducted at Oslo University Hospital in Norway from 2010 to 2014. Participants with BPD (n = 25) were recruited from the Department of Personality Psychiatry (DPP), whereas participants with SZ (*n* = 25) and healthy control participants (HC, *n* = 25) were recruited from the Thematically Organized Psychosis (TOP) Study at NORMENT KG Jebsen Centre for Psychosis Research. The regular staff at DPP conducted the clinical interviews of participants with BPD. Diagnoses were based on the DSM-IV criteria using The Structured Clinical Interview for DSM-IV Axis II Disorders (SCID-II; First, 1994). Within TOP, clinical psychologists or medical doctors who have participated in an international training program (Ventura et al., 1998) undertake the diagnostic and clinical assessments. They receive supervision, and diagnoses are largely consensus-based. The Structured Clinical Interview for DSM-IV Axis I Disorders (SCID-I; First et al., 1995) is used for diagnostic purposes. HCs from the same geographical areas were recruited through national statistical records and invited by letter to participate. They were screened with an interview to capture symptoms of severe mental illness (Primary Care Evaluation of Mental disorders; PRIME-MD; Spitzer et al., 1994) and excluded if there was any information on mental, neurological or somatic disorder. Only participants with excellent knowledge of Norwegian were included with participants of non-Norwegian descent being required to have lived in Norway for the last 10 years. The Regional Ethics Committee South East and the Norwegian Data Inspectorate approved the study. All participants received oral and written information on the study and have signed informed consent. All participants were female. The demographics of the three groups are presented in Table 1. One-way univariate analyses of variance (ANOVAs) showed a statistically significant group difference for education (women with SZ had significantly less education than healthy women), but not for age.

**TABLE 1:** **Demographic characteristics in women with borderline personality disorder or schizophrenia, and in healthy women. Clinical characteristics in women with borderline personality disorder or schizophrenia**.

	**BPD *n* = 25 Mean (SD)**	**SZ *n* = 25 Mean (SD)**	**HC *n* = 25 Mean (SD)**	**Statistic**
Age	30.7 (5.9)	30.8 (10.0)	30.6 (8.6)	*F* (2,74) < 0.01, *p* = 0.995
Education (years)	13.6 (2.7)	12.5 (2.2)	14.3 (2.4)	*F* (2,74) = 3.56, *p* = 0.034
GAF-s	48.4 (4.6)	43.5 (11.8)	–	*t* = –1.92, *p* = 0.060
GAF-f	48.7 (5.4)	45.6 (11.6)	–	*t* = –1.20, *p* = 0.236
PANSS-pos	–	14.9 (4.2)	–	–
PANSS-neg	–	12.5 (3.2)	–	–

BPD, personality disorder; SZ, schizophrenia; HC, healthy controls; SD, standard deviation; GAF-s, Global Assessment of Functioning symptoms; GAF-f: Global Assessment of Functioning, PANSS, Positive and Negative Syndrome Scale, pos, positive symptoms, neg, negative symptoms.

### Measure of Social Cognition

**FIGURE 1:**
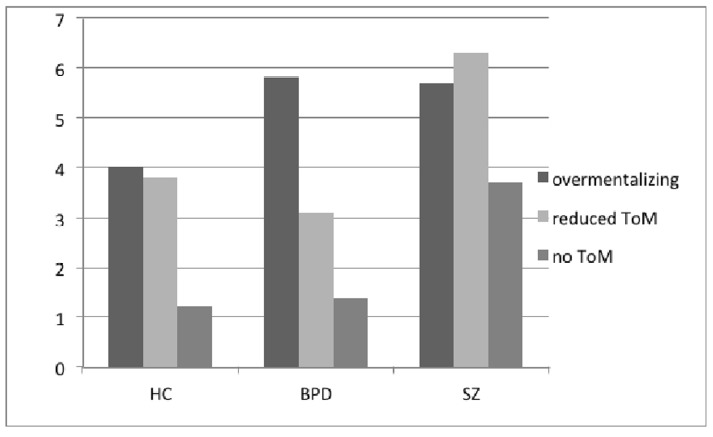
**ToM error patterns in healthy women, women with borderline personality disorder, and in women with schizophrenia.** ToM, theory of mind; HC, healthy controls; BPD, borderline personality disorder, SZ, schizophrenia.

Social cognition was indexed by ToM and assessed with the Norwegian version (Fretland et al., 2015) of the MASC (Dziobek et al., 2006). The test consists of a 15-min video showing four people that come together for dinner and can be considered an ecologically valid measure (Dziobek et al., 2006). The video is stopped 45 times for the test taker to answer a question about the thoughts, emotions or intentions of one of the characters using a multiple-choice format. The multiple-choice format is comprised of four answers where one is correct and three are wrong. The three error types are overmentalizing, reduced ToM and no ToM, where the two latter are types of undermentalizing. Overmentalizing means excessively attributing intentions or personal meaning, undermentalizing refers to a lack of functional concepts of mental state. Reduced ToM means that a person is capable of mentalizing, but does it incorrectly, whereas no ToM indicates lack of mentalizing ability. To ensure construct validity of overmentalizing answers, care was taken during test development to make sure that the complexity of the answer referred to the mental state of the character (and not to complex linguistics). Empirical support for the construct validity comes from previous studies that found associations between overmentalizing and positive symptoms (Montag et al., 2011; Fretland et al., 2015). Data is presented in Table 2 and Figure 1, including standardized scores (*z*-scores; mean = 0, standard deviation = 1) based on the mean and standard deviation of the HCs.

**TABLE 2:** **Theory of mind in women with borderline personality disorder or schizophrenia, and in healthy women**.

	**HC *n* = 25**		**BPD *n* = 25**		**SZ *n* = 25**		**Statistic**
	**Mean (SD)**	**z**	**Mean (SD)**	**z **	**Mean (SD)**	**z **	
MASC total correct	36.0 (3.6)	0	34.7 (4.5)	–0.37	29.2 (6.4)	–1.87	ANOVA: *F* = 12.9** SZ < BPD, HC
MASC overmentalizing errors	4.0 (2.2)	0	5.8 (3.5)	0.83	5.7 (4.1)	0.78	Two-way mixed ANOVA: Group: *F* = 12.5**
MASC “reduced ToM” errors	3.8 (1.8)	0	3.1 (2.1)	–0.42	6.3 (3.3)	1.38	Error: 29.2**
MASC “no ToM” errors	1.3 (1.4)	0	1.4 (1.5)	0.06	3.7 (2.5)	1.74	Error × group: 3.3*

HC, healthy controls; BPD, personality disorder; SZ, schizophrenia; SD, standard deviation; z, standardized score; MASC, Movie for the Assessment of Social Cognition; ToM, theory of mind; SZ, schizophrenia; BPD, borderline personality disorder; HC, healthy control participants, **p < 0.001; *p < 0.05.

### Clinical Measures

Global functioning was assessed with the Global Assessment of Functioning Scale-split version (GAF; Pedersen et al., 2007). The split into one symptom (GAF-s) and one function score (GAF-f) is a means to improve the psychometric properties of the scale and our reason for choosing this version. GAF scores are presented in Table 1. The two clinical groups did not differ significantly in global symptoms or global functioning. Psychotic symptoms in the SZ group were assessed with the positive and negative syndrome scale (PANSS; Kay et al., 1987). See Table 1 for symptom scores. Values indicate that participants were in a clinically stable state at time of assessment.

### Statistical Analyses

All analyses were computed using The Statistical Package for the Social Sciences (IBM SPSS Statistics for Windows, version 22.0, IBM Corp, Armonk, NY, USA). The first research aim concerning group differences in overall ToM ability was investigated using a univariate analysis of variance (ANOVA) with *post hoc* Scheffé comparisons. The total MASC score was entered as the dependent variable and diagnostic group (BPD/SZ/HC) as the independent variable. For our second research aim a two-way mixed ANOVA was conducted with one within-subjects factor (the three MASC error scores: overmentalizing/reduced ToM/no ToM) and one between-subjects factor (diagnostic group: BPD/SZ/HC). A significant error type × diagnostic group interaction will indicate that error patterns depend on group membership.

## Results

The initial ANOVA revealed a significant group difference in overall ToM ability [*F*(2,75) = 12.93, *p* < 0.001, η^2^ = 0.26]. See Table 2. This effect did not change when controlling for education [*F*(2,75) = 12.06, *p* < 0.001, η^2^ = 0.25], and education was therefore not controlled for in subsequent analyses. Scheffé *post hoc* analyses revealed that it was the SZ group that differed significantly, with lower overall score, compared to the other two groups. In the two-way mixed ANOVA, the main between-subject effect of diagnostic group [*F*(2,71) = 12.51, *p* < 0.001, η^2^ = 0.68] was significant. Mauchly’s test indicated that the assumption of sphericity was violated (x^2^ (2) = 48.6, *p* < 0.001), and degrees of freedom were consequently corrected using Greenhouse-Geisser estimates of sphericity. The main within-subjects effect of error type was significant [*F*(1.337,96.260) = 29.2, *p* < 0.001, η^2^ = 0.29], as was the error type × diagnostic group interaction effect [*F*(2.674, 96.260) = 3.3, *p* = 0.028, η^2^ = 0.08]. See Table 2 and Figure 1. This points to differences in error patterns between groups. Women with SZ committed more of all error types compared to healthy women. Women with BPD made more overmentalizing errors than healthy women, on a level comparable to women with SZ. The *z*-scores in Table 2 are, as expected, in line with these results.

## Discussion

In this study we compared ToM abilities in women with BPD and SZ. One of the main findings is that women with BPD were not impaired in overall ToM abilities. They did not perform significantly different compared to our healthy female control group. This was in stark contrast to women with SZ whose overall ToM score fell markedly below that of the healthy women. From the *z*-scores we see that the SZ group performed close to two standard deviations (*z* = –1.87) below the HC, which indicates a very large effect size. It is in fact larger than the effect size from a meta-analysis of the ToM SZ literature (Hedge’s *g* = 0.96; Savla et al., 2013), possibly attributable to ToM having been assessed with simpler tasks in several of the studies included in that meta-analysis. A previous study found superior mentalizing abilities in women compared to males with SZ (Abu-Akel and Bo, 2013). Our study indicates that female gender is not a protection against impaired social cognition in individuals with SZ. In a previous study we found no gender differences for overall ToM performance in individuals with SZ using the MASC test (Fretland et al., 2015). Taken together, our findings suggest that women with SZ do not have superior mentalizing abilities compared to males with SZ or to healthy women. However, more studies including men with SZ are necessary to make firm conclusions regarding gender differences in ToM abilities. The same is the case for men with BPD, but based on the current study and the literature on BPD gender differences in general (Silberschmidt et al., 2015), we would expect intact ToM as assessed with the MASC even for men with BPD.

Although women with BPD were not significantly impaired compared to HC, their *z*-score for overall ToM (–0.37) approached half a standard deviation below HC. Within a clinical neuropsychological perspective this score may be interpreted as subtle social cognitive problems. It was therefore of interest to compare the error patterns in our three study groups. First of all, there were differences between error types, meaning that some of them were committed more often than others. However, the exact distribution of error types differed between the three study groups. Referring to Figure 1, we see that “no ToM” was the rarest occurring error in all three groups; hardly ever seen in the BPD (*z* = 0.06) and HC groups. Women with SZ made more “no ToM” (*z* = 1.74) errors than the other two study groups. They did, however, make numerically more overmentalizing and “reduced ToM” errors, of approximately the same amount. For these two error types the largest difference, compared to the HC group, was seen for “reduced ToM” errors (*z* = 1.38). The BPD group made fewer “reduced ToM” errors than HC (*z* = –0.42), but more overmentalizing errors (*z* = 0.83), actually slightly more so than women with SZ did compared to HC (*z* = 0.78). This paints an interesting picture of possible differences in social cognition in BPD and SZ.

Whereas BPD does not seem to be characterized by prominent difficulties in ToM, our results suggest that subtle impairments may be present. Based on this study, it seems that women with BPD do not make a lot of mistakes when interpreting the thoughts, emotions and intentions of others. But when they do, they make overmentalizing errors. These findings align with the older literature on mentalizing in BPD where it was claimed that this population has a “hypervigilant” mind. Sensitivity to social cues can transfer to a mentalizing style of overly attributing intentions, i.e., committing overmentalizing errors. Such a mentalizing style can further down the line contribute to the interpersonal problems seen for individuals with BPD. Moreover, the present results are in line with the findings of Sharp et al. (2011, 2013) from an adolescent sample indicating a particular relationship between BPD traits and overmentalizing errors. The present study is the first to report overmentalizing as a characteristic feature of ToM performance errors in adult women with BPD.

Women with SZ had large impairments in ToM assessed with an ecologically valid test. As predicted, they made both overmentalizing and undermentalizing errors. The fact that their errors were not limited to “no ToM” responses is evidence that they indeed have a ToM, but that they often use it incorrectly. In other words, there is no reason to claim that SZ is characterized by so-called “mind-blindness.” Instead their mentalizing style is not as specific as seems to be the case for BPD.

One may speculate that there are different reasons for the overmentalizing errors (level of which was identical) in our two clinical groups. We have previously shown, in a partly overlapping sample with the inclusion of males with SZ, that positive symptoms such as hallucinations and delusions had small-to-moderate, albeit statistically significant associations with overmentalizing in participants with SZ (Fretland et al., 2015). Delusions involve drawing conclusions and inferences that are false and not based on existing evidence. Overmentalizing is a similar process where inferences are exaggerated compared with evidence that is in fact present. It would not be surprising if these processes were somewhat linked, and we find it likely that this is what is at play for SZ. For BPD, which is not characterized by positive psychotic symptoms, the explanation must be another. It is has been shown that comorbid post-traumatic stress disorder or a history of sexual trauma is associated with increased ToM deficits in BPD (Fonagy et al., 1996; Preißler et al., 2010). These are prevalent conditions in individuals with BPD (Yen et al., 2002). A history with exposure to life-threatening situations of abuse can render a person extremely sensitive to social cues and possibly to a mentalizing style of overly attributing (bad) intentions to other people. The relationship between trauma and ToM is probably complex. Whereas parental under-involvement and abuse may hamper the development of ToM (Fonagy et al., 1996), studies of mentalizing deficits, operationalized as Reflective Functioning, indicate that mentalizing capacity may further mediate the relationship between childhood adversities and adult BPD (Chiesa and Fonagy, 2014; Katznelson, 2014). Thus, intact ToM could also serve as a buffer against the detrimental effects of trauma. Moreover, Sharp et al. (2011) found that the association between overmentalizing, as assessed by MASC, and BPD traits was mediated by emotional dysregulation, suggesting that such ToM errors interfere with the ability to regulate emotions within an interpersonal context. Although we are unable to test these hypotheses within the current study, we propose that overmentalizing errors have slightly different origins in these two disorders.

It should also be noted that it is possible that the ToM abilities of individuals with BPD may be compromised only in situations where their attachment system is activated (Fonagy and Higgitt, 1990). Such activation is likely to be characterized by emotional arousal, which is thought to alter mentalizing capacity in persons with BPD (Fonagy and Luyten, 2009). Following this line of thinking, ToM skills would be intact in emotionally neutral situations, such as when solving the MASC test, but would break down in an emotionally charged situation that activates insecure or disorganized attachment models. Empirical testing of this hypothesis requires ToM tests with emotional or personally activating material.

Limitations of the study are for one that we were not able to include males, due to few males presenting with BPD at the Department of Personality Psychiatry. Therefore, our results must be viewed with caution and cannot be generalized to the whole of the BPD/SZ populations. A second limitation is the lack of instruments and information for both clinical groups besides social cognition and global functioning. This is due to the fact that this is a joint study of two research laboratories at Oslo University Hospital with different traditions and focus areas. Future studies would benefit from the inclusion of information collected with the same instruments for all included participants. This would both increase the depth of comparisons of two mental disorders that in many ways are different, as well as yield more information on the possible clinical implications across diagnostic groups. Another future direction worth pursuing would be to examine if and how history of traumatization, including abuse and neglect, relates to social cognition/ToM for both SZ and for BPD. Interestingly, it seems that ToM can be largely intact, but still be associated with the experience of early adversity, as shown in a study of BPD using a ToM cartoon task (Ghiassi et al., 2010).

In summary, this study found large ToM impairments for SZ with a mentalizing style of both under- and overmentalizing. BPD presented with intact overall ToM ability, but with a mentalizing style of overly attributing thoughts, emotions and intentions.

## Author Contributions

AV designed the study, translated the ToM measure into Norwegian, collected data, conducted the statistical analyses, interpreted the data and drafted the first version of the manuscript. BTA designed the study, collected data, and contributed to interpretation of results and text revisions. RAF designed the study, collected data and contributed to interpretation of results. KS designed the study and contributed to statistical analyses and to interpretation of results. ID developed the original version of the ToM measure, designed the study and contributed with interpretation of the findings. TW designed the study and contributed to interpretation of results and text revisions. All authors have revised the manuscript for important intellectual content, have approved the final version and agree to be accountable for all aspects of the work.

### Conflict of Interest Statement

The authors declare that the research was conducted in the absence of any commercial or financial relationships that could be construed as a potential conflict of interest.
